# Pathological presentation of cardiac mitochondria in a rat model for chronic kidney disease

**DOI:** 10.1371/journal.pone.0198196

**Published:** 2018-06-11

**Authors:** Einat Bigelman, Lena Cohen, Genya Aharon-Hananel, Ran Levy, Zach Rozenbaum, Ann Saada, Gad Keren, Michal Entin-Meer

**Affiliations:** 1 Sackler School of Medicine, Tel-Aviv University, Tel-Aviv, Israel; 2 The Laboratory of Cardiovascular Research, Department of Cardiology, Tel-Aviv Sourasky Medical Center, Tel-Aviv, Israel; 3 Internal Medicine Unit T, Tel-Aviv Sourasky Medical Center, Tel-Aviv, Israel; 4 Department of Genetic and Metabolic Diseases, Hadassah-Hebrew University Medical Center, Jerusalem, Israel; University Medical Center Utrecht, NETHERLANDS

## Abstract

**Background:**

Mitochondria hold crucial importance in organs with high energy demand especially the heart. We investigated whether chronic kidney disease (CKD), which eventually culminates in cardiorenal syndrome, could affect cardiac mitochondria and assessed the potential involvement of angiotensin II (AngII) in the process.

**Methods:**

Male Lewis rats underwent 5/6 nephrectomy allowing CKD development for eight months or for eleven weeks. Short-term CKD rats were administered with AngII receptor blocker (ARB). Cardiac function was assessed by echocardiography and cardiac sections were evaluated for interstitial fibrosis and cardiomyocytes’ hypertrophy. Electron microscopy was used to explore the spatial organization of the cardiomyocytes. Expression levels of mitochondrial content and activity markers were tested in order to delineate the underlying mechanisms for mitochondrial pathology in the CKD setting with or without ARB administration.

**Results:**

CKD *per-se* resulted in induced cardiac interstitial fibrosis and cardiomyocytes’ hypertrophy combined with a marked disruption of the mitochondrial structure. Moreover, CKD led to enhanced cytochrome C leakage to the cytosol and to enhanced PARP-1 cleavage which are associated with cellular apoptosis. ARB treatment did not improve kidney function but markedly reduced left ventricular mass, cardiomyocytes’ hypertrophy and interstitial fibrosis. Interestingly, ARB administration improved the spatial organization of cardiac mitochondria and reduced their increased volume compared to untreated CKD animals. Nevertheless, ARB did not improve mitochondrial content, mitochondrial biogenesis or the respiratory enzyme activity. ARB mildly upregulated protein levels of mitochondrial fusion-related proteins.

**Conclusions:**

CKD results in cardiac pathological changes combined with mitochondrial damage and elevated apoptotic markers. We anticipate that the increased mitochondrial volume mainly represents mitochondrial swelling that occurs during the pathological process of cardiac hypertrophy. Chronic administration of ARB may improve the pathological appearance of the heart. Further recognition of the molecular pathways leading to mitochondrial insult and appropriate intervention is of crucial importance.

## Introduction

Kidney dysfunction and cardiac dysfunction are frequently associated, leading to cardiorenal syndrome (CRS)—a grave condition with growing morbidity and mortality rate. Two main known pathways may connect renal and cardiac dysfunctions: one is the pro-inflammatory response which positively correlates with the severity of the disease;[1–3] and the second is the inappropriate upregulation of the Renin Angiotensin System (RAS), a major hallmark of both chronic kidney disease (CKD) and chronic heart failure.[4]

CRS is composed of five sub-types according to the different pathogenesis.[5] Among which, CRS type 4 is characterized by underlying CKD which leads to an impairment of cardiac function, left ventricular hypertrophy (LVH), diastolic dysfunction, and/or increased risk of adverse cardiovascular events.[6]

Recently, it has become clear that cardiovascular involvement occurs in each stage of CKD and that major cardiac events actually represent almost 50% of the causes of death in CKD patients.[7] Moreover, the tight interplay between kidney and heart exposes individuals with a relatively minor reduction in estimated glomerular filtration rate to an increased risk of cardiovascular events and death.[5]

We have postulated that mitochondria, which hold crucial importance in the heart due to its high-energy demand, may be implicated in CRS. Mitochondria are known to be involved in crucial metabolic processes including oxidative phosphorylation, steroid and heme biosynthesis, intermediary metabolism, calcium and iron homeostasis, programmed cell death and innate immunity.[8, 9] In this regard, numerous cardiovascular diseases have been associated with mitochondrial dysfunction linked to mitochondrial swelling and lysis; among which, cardiomyopathy [10, 11] as well as cardiac hypertrophy, ischemia/reperfusion and heart failure.[12–15]

In previous studies performed in our laboratory we have looked into the molecular mechanisms which may lead to cardiac disease progression in short-term CKD rat model.[16–18] Interestingly, Gene Chip expression array analysis showed that CKD *per-se* attenuates expression levels of several key genes of cardiac mitochondria, especially genes involved in the ubiquinone biosynthesis pathway. One of these genes whose expression was downregulated is ubiquinone, also known as coenzyme Q10, which is present primarily in the mitochondria and serves as a critical component of the energy-generating electron transport chain.

Nevertheless, to date, the mechanisms leading from CKD to CRS are not completely deciphered, and in particular, the molecular pathways connecting CKD to cardiac mitochondria alterations are not well established. In the current study we sought to assess the mitochondrial structure and function of cardiac tissue in the setting of CKD using a rat model for 5/6 nephrectomy. The data presented herein demonstrate that the state of CKD manifests into cardiac pathology that includes interstitial fibrosis, cardiomyocytes hypertrophy and mitochondrial swelling and damage. These pathological changes could be partially reversed following treatment with Angiotensin II (AngII) receptor blocker (ARB). The data shed light on cardiac mitochondria as a potential target in the treatment of CKD patients and point to the crucial significance of ARB administration in early stages of CKD in order to attenuate cardiac mitochondrial alterations in CKD patients.

## Materials and methods

### Ethics statement

Animal studies were approved by The Animal Care and Use Committee of Tel Aviv Sourasky Medical-Center (approval number 8n-5-15) which conforms to the policies of the American Heart Association and the Guide for the Care and Use of Laboratory Animals.

### Animals

Male Lewis rats (250–350 gr) were purchased from Envigo, Israel. All rats were kept under optimal conditions (food and water provided *ad libitum*) at room temperature in a temperature-controlled facility with a 12 hrs. light/dark cycle. Prior to all surgical procedures, animals were anesthetized with a mixture of ketamine/xylazine (100/10 mg/kg, respectively) and anesthesia was confirmed by loss of pedal reflex (toe pinch). During experimental periods, animals were monitored 2–3 times per week for potential signs of suffering, mainly weight loss of more than 15% and significant changes in animals’ behavior, mobility or body posture. Should rats have met one of these criteria; euthanasia would have been warranted on the same day in order to prevent further suffering.

### Rat model for CKD

Rats underwent 5/6 nephrectomy for induction of CKD as previously established in our laboratory.[16] Briefly, 5/6 nephrectomy was performed in two consecutive surgeries: two-thirds of the left kidney was removed followed by removal of the right kidney a week later. Control animals underwent abdominal opening only (Sham). Animals were kept for eight months. Two animals died during the echocardiography procedure: one from each group. In addition, during the long-term CKD period, one rat was euthanized due to loss of more than 15% of its body weight and 4 other rats died in the late experimental period (after four to seven months); therefore, the final total number of animals was 15 in CKD and 17 in sham groups. Urine samples were collected for 24-hour time periods, during which the animals were kept in metabolic cages (Lavotal, Italy) where they were allowed to eat and drink *ad libitum*. Blood and urine samples were collected in order to determine plasma levels of AngII, pro- and anti-inflammatory cytokines, Blood Urea Nitrogen (BUN) and to calculate creatinine clearance (Ccr).[16] At time of necropsy, heart and kidney weights were documented by weighing the organs immediately after excision. Tissues were kept at -80°C for further analysis.

### Treatment with ARB

In a new set of experiment for short-term CKD model, one day after the second surgery, nephrectomized and sham rats were randomly divided into 3 groups (n = 7/arm) and treated for a total period of 11 weeks as followed: (1) sham-operated control (Sham group); (2) 5/6 nephrectomized (CKD group) rats were administrated with regular drinking water; (3) 5/6 nephrectomized rats administrated with Losartan, an AngII receptor blocker (ARB) (100mg/500ml) in drinking water (ARB group). In order to evaluate whether ARB affects cardiac mitochondria, additional group of sham-operated control rats were administrated with Losartan (100mg/500ml) in drinking water (Sham-ARB group). These results are detailed in supporting information S1 and S2 Tables. No mortality was evident prior to termination of the short-term experiment.

### Blood pressure

One week prior to termination of the experiment, repeated blood pressure (BP) measurements were taken using 3-channel computerized tail-cuff IITC system model 3M229 BP, attached to the 31BP software package (IITC Life Science Inc, USA).

### Echocardiography

Cardiac function was evaluated one day prior to termination by transthoracic echocardiography (Vevo 2100, VisualSonics, Toronto, Canada) as described previously.[1, 17, 18] Left ventricular end-diastolic diameter (LVEDD), left ventricular end-systolic diameter (LVESD), intraventricular septal wall thickness (IVSd) and posterior wall (PWd) thickness were measured in systole and diastole to determine the presence of hypertrophy. Assuming that the rat’s LV geometry is sphere-shaped, the LV mass was calculated according to the following formula:

$$LV\;mass={1.047\times (LVEDD+PWd+IVSd)}^{3}-{LVEDD}^{3}$$

The value 1.047 represent the specific gravity of muscle, and the ratio (LV mass/body weight) was defined as the hypertrophy ventricular index (mg/gr).[19]

### Histology & immunohistochemistry of LV sections

By the end of the experiments, animals were scarified and heart tissues were isolated and fixed with 4% paraformaldehyde. LV samples were then sliced into transverse sections and paraffinized. The blocks were sectioned at 5 μm thick slices. In order to assess cardiac fibrosis as percent of fibrosis out of total area, LV sections were stained with Masson’s trichrome (Sigma, St. Louis, MO) and viewed by a light microscope. Cardiomyocytes’ hypertrophy was monitored by FITC-wheat germ agglutinin (WGA) staining and viewed by fluorescent microscopy (Olympus BX 51). LC3B (microtubule-associated protein light chain 3, isoform B) was detected using rabbit anti-LC3B polyclonal antibody (Novus Biological) with hematoxylin counter staining according to manufacturer’s instructions.

### Transmission electron microscopy

At time of necropsy, 1mm^2^ of cardiac tissue samples were fixated in 2.5% Glutaraldehyde for Transmission Electron Microscope (TEM) processing according to standard protocol. Thin sections were viewed and photographed by TEM (JEOL 1010). Cardiac mitochondrial volume was measured as percentage of mitochondrial area out of total area in each measured field, regardless of the total number of cardiomyocytes in each particular field. The image field is given at a 12K magnification.

### Image analysis

Quantification analysis of cardiac fibrosis, cardiomyocytes’ hypertrophy and cardiac mitochondrial volume was performed by the Image J software.

### Western blot

Heart samples were homogenized using a commercial lysis buffer (Sigma) for whole tissue lysates or underwent cytosolic/mitochondrial fractionation using commercial kit (BioVision, Inc.) according to the manufacturer’s instructions. Equal protein amounts (12.5μg for cytosolic fractions and 50μg for whole tissue lysates) were loaded on 4–15% gradient acrylamide gels and subjected to Western blot as previously described.[20] The membranes were incubated with primary antibodies of anti-: Peroxisome proliferator-activated receptor gamma coactivator 1-alpha (PGC1α), Angiotensin II type 1 receptor (AT1R), PINK1, GAPDH (Abcam); PARP1 (Santa Cruz); cytosolic cytochrome C (CytC), mitochondrial MFN1, mitochondrial DRP1 (Bios); or with mitochondrial FIS1 (MBL). The membranes were then incubated with HRP-conjugated secondary antibodies. After rapid incubation with an ECL substrate (Biological Industries, Israel) the membranes were exposed to an imaging film. When mitochondrial fractions were tested, Ponceau staining was used to normalize for equal protein loading of the gels.

### Quantitative real-time polymerase chain reaction

Total RNA was extracted using phenol/chloroform EZ-RNA kit (Biological Industries, Israel). RNA (500ng/reaction) was reverse-transcribed to cDNA using Verso cDNA Kit (Thermo Scientific). Quantitative real-time polymerase chain reaction (PCR) using SYBR^®^ Green PCR Master Mix (Applied Biosystems) was performed with specific primers to the gene of interest (Sigma-Aldrich Israel Ltd.).

The sequences of Cytochrome B (CytB) primers used were: forward 5′-TGACCTTCCCGCCCCATCCA-3′ and revers 5′-AGCCGTAGTTTACGTCTCGGCA-3′; and was normalized to the expression of the 18S reference gene (forward 5′-TTGATTAAGTCCCTGCCCTTT-3′ and revers 5'-CGATCCGAGGGCCTAACTA-3').

Product purity was validated by a melt curve with ABI hardware and software (StepOne RT-PCR) and analyses of gene expression were derived by the comparative ΔΔCt method.[20]

### Functional assays of the mitochondria

LV lysates obtained from experimental rats were used to assess the content of intact mitochondria via measurement of the enzymatic activity of citrate synthase (CS), a mitochondrial marker enzyme localized in mitochondrial matrix.[21, 22] Likewise, mitochondrial ATP synthase specific activity was measured.

CS activity (Sigma-Aldrich Co. LLC) and specific enzymatic activity of ATP synthase (Abcam) were measured using commercial kits in accordance to manufacturer instructions.

### Blood levels of AngII and cytokines

Plasma levels of AngII were measured using AngII enzyme immunoassay (EIA) kit (RayBio^®^) according to the manufacturer’s instructions. Serum cytokine profile of IL-1α, IL-1β, TNFα, IFNγ, IL-2, IL-4, IL-10, IL-13 and MCP-1 was determined by cytokine microarray analysis using a commercially available array kit (Quantibody^®^ Rat Inflammation Array) according to the manufacturer’s instructions.

### Statistical analysis

SPSS (IBM^®^ SPSS^®^ Statistics; Version 22) was used for statistical analysis. All variables are expressed as means ± standard error of mean (SEM). The *Shapiro-Wilk’s test* was used to evaluate Gaussian distribution of all parameters. *The two-tailed Student t-test* (if data were normally distributed) or *Mann-Whitney test* (if data were not normally distributed) were used to compare between two groups. *One way ANOVA followed by Tukey’s post-hoc test* (if data were normally distributed) or *Kruskal–Wallis H test* (if data were not normally distributed) were used to compare between three groups. In all tests, p< 0.05 was considered statistically significant. A “trend” towards significance (T) was considered to be 0.05<p<0.1. P^A^ represent the p-value of non-parametric analysis (*Mann-Whitney test* or *Kruskal–Wallis H test)* when data did not normally distributed.

## Results

### Hemodynamic, heart and kidney parameters in CKD

First, we thoroughly characterized renal, cardiac and hemodynamic parameters in the long-term CKD model (Table 1). As expected, body weight and creatinine clearance (Ccr) was decreased and sera BUN was increased in the CKD group compared to sham-operated control, validating the deteriorating kidney function. The weight of the remaining kidney as well as the ratio of kidney/body weight was markedly increased in the CKD setting relative to sham, as previously documented by us as well as by others.[16, 23] These changes were accompanied by a mild increase in BP. Concomitantly, the CKD group exhibited significant alterations in cardiac morphology. Echocardiography test demonstrated an increase in parameters of cardiac hypertrophy including LV mass, hypertrophy ventricular index, relative wall thickness, intraventricular septum diastole and LV posterior wall thickness in diastole though systolic function represented by ejection fraction and fractional shortening has not yet deteriorated. The data suggest that long-term CKD *per-se* leads to cardiac hypertrophy, without an apparent reduction in cardiac function.

**Table 1:** Hemodynamic, heart and kidney parameters in long-term CKD versus sham rats.

	Sham	CKD		p-value
**Blood pressure**				
Systolic blood pressure (mmHg)	119±2.4 (n = 8)	**↑**	**134±2.5 (n = 9)**	**p = 0.001**
Diastolic blood pressure (mmHg)	73±4.9 (n = 8)	-	80±5.2 (n = 9)	NS (0.33)
**Renal parameters**				
Body weight (gr)	513±22 (n = 17)	**↓**	**436±13 (n = 15)**	**p**^**A**^ **= 0.027**
Kidney weight (gr)	1.33±0.03 (n = 17)	**↑**	**1.63±0.05 (n = 15)**	**p<0.001**
Kidney/Body weight (mg/gr)	2.68±0.15 (n = 17)	**↑**	**3.74±0.11 (n = 15)**	**p<0.001**
Plasma BUN (mg/dL)	15.11±0.54 (n = 9)	**↑**	**55.55±5.57 (n = 9)**	**p<0.001**
Plasma creatinine (mg/dL)	0.43±0.04 (n = 9)	**↑**	**1.05±0.12 (n = 9)**	**p**^**A**^**<0.001**
Creatinine clearance (ml/min)	1.04±0.15 (n = 5)	**↓**	**0.48±0.08 (n = 5)**	**p = 0.014**
Urinary albumin excretion rate (mg/min)	0.14±0.04 (n = 5)	**↑**	**4.77±0.68 (n = 5)**	**p = 0.002**
Urinary protein excretion rate (mg/min)	0.29±0.05 (n = 5)	**↑**	**4.17±1.07 (n = 5)**	**p = 0.022**
**Cardiac parameters**				
LV mass (mg)	585±55 (n = 9)	**↑**	**824±73 (n = 9)**	**p = 0.018**
Hypertrophy ventricular index (mg/gr)	1.26±0.12 (n = 8)	**↑**	**2.01±0.18 (n = 8)**	**p = 0.004**
Relative wall thickness (RWT)	0.57±0.03 (n = 9)	**↑**	**0.72±0.04 (n = 9)**	**p = 0.018**
Fractional shortening (FS, %)	44±2.9 (n = 9)	-	53±5.3 (n = 9)	NS (0.23)
Ejection fraction (EF)	79±2.6 (n = 9)		85±3.9 (n = 9)	NS (0.23)
Intraventricular Septum Diastole (mm)	1.55±0.08 (n = 9)	**↑**	**1.84±0.08 (n = 9)**	**p = 0.024**
LV Posterior Wall Thickness in Diastole (mm)	1.68±0.08 (n = 9)	**↑**	**2.13±0.04 (n = 9)**	**p = 0.002**
LV—end diastolic diameter (LVEDD) (mm)	5.9±0.21 (n = 9)	-	6.02±0.33 (n = 9)	NS (0.71)
LV—end systolic diameter (LVESD) (mm)	3.4±0.22 (n = 9)	-	2.8±0.38 (n = 9)	NS (0.26)

LV- left ventricle, BUN- blood urea nitrogen, NS- non significant;

p^A^ is based on Mann-Whitney test, otherwise p-value is based on *two-tailed t-test*.

### Cytokines and Angiotensin II involvement in the setting of CKD

**Fig 1:**
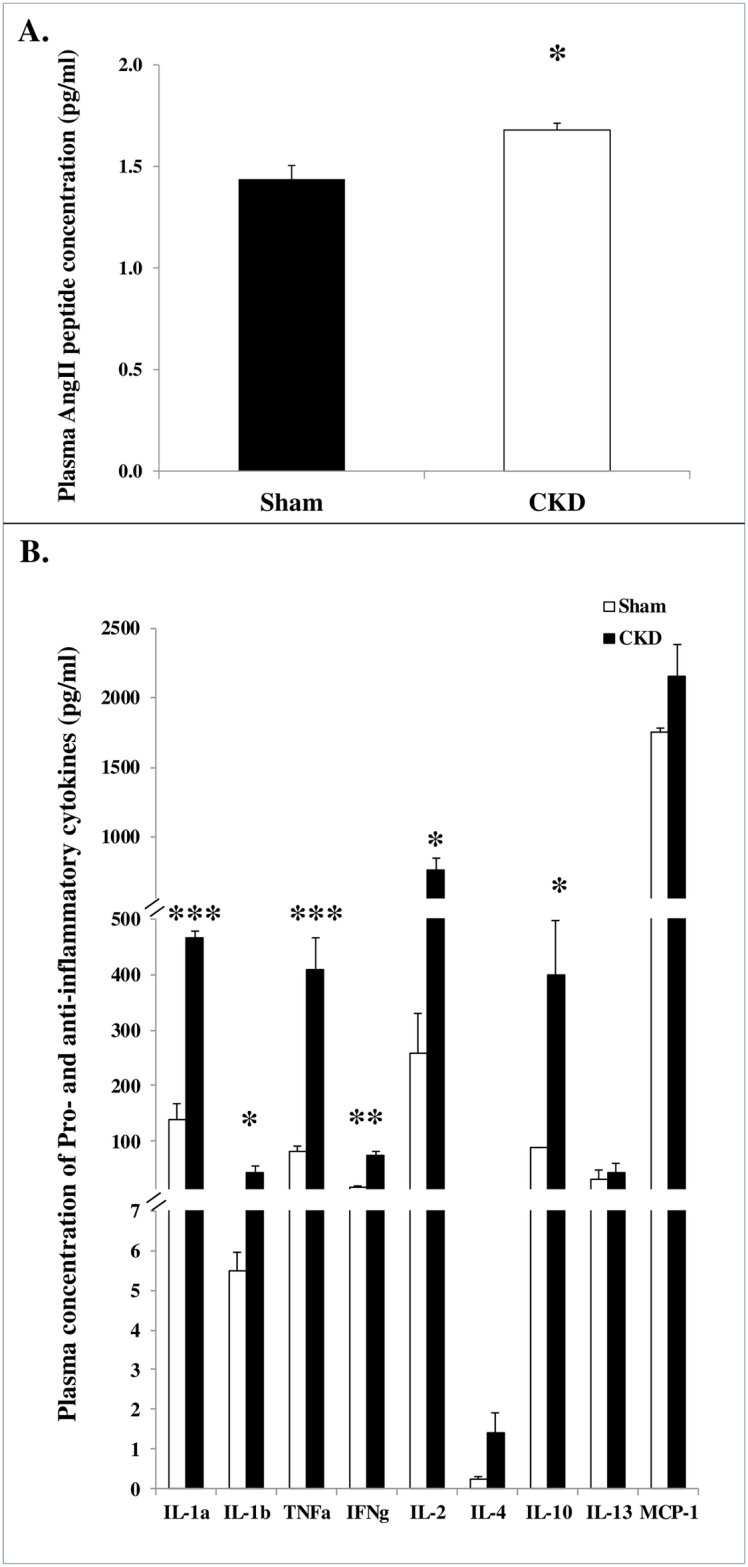
CKD-mediated systemic effects on the neurohumoral and immune systems. Plasma levels of Angiotensin II **(A)**; and Pro- and anti-inflammatory cytokine profile **(B)** in plasma samples of CKD rats (n = 3) compared to sham (n = 4). Data are represented as mean ± SEM. ** p<0.01,* p<0.05.

Next, we assessed the systemic effects of CKD setting on the neurohumoral response as well as on the cytokine profile. As expected, a slight but statistically significant increase was documented in AngII plasma levels in the CKD group relative to sham (1.68±0.03pg/ml versus 1.44±0.07pg/ml, respectively; p = 0.035; Fig 1A). In addition, levels of IL-1α, IL-1β TNFα, INFγ, IL-2 and IL-10 were elevated in the sera of CKD compared to sham (Fig 1B), suggesting that the chronic state of renal disease affects the inflammatory network.

### Cardiac histology

**Fig 2:**
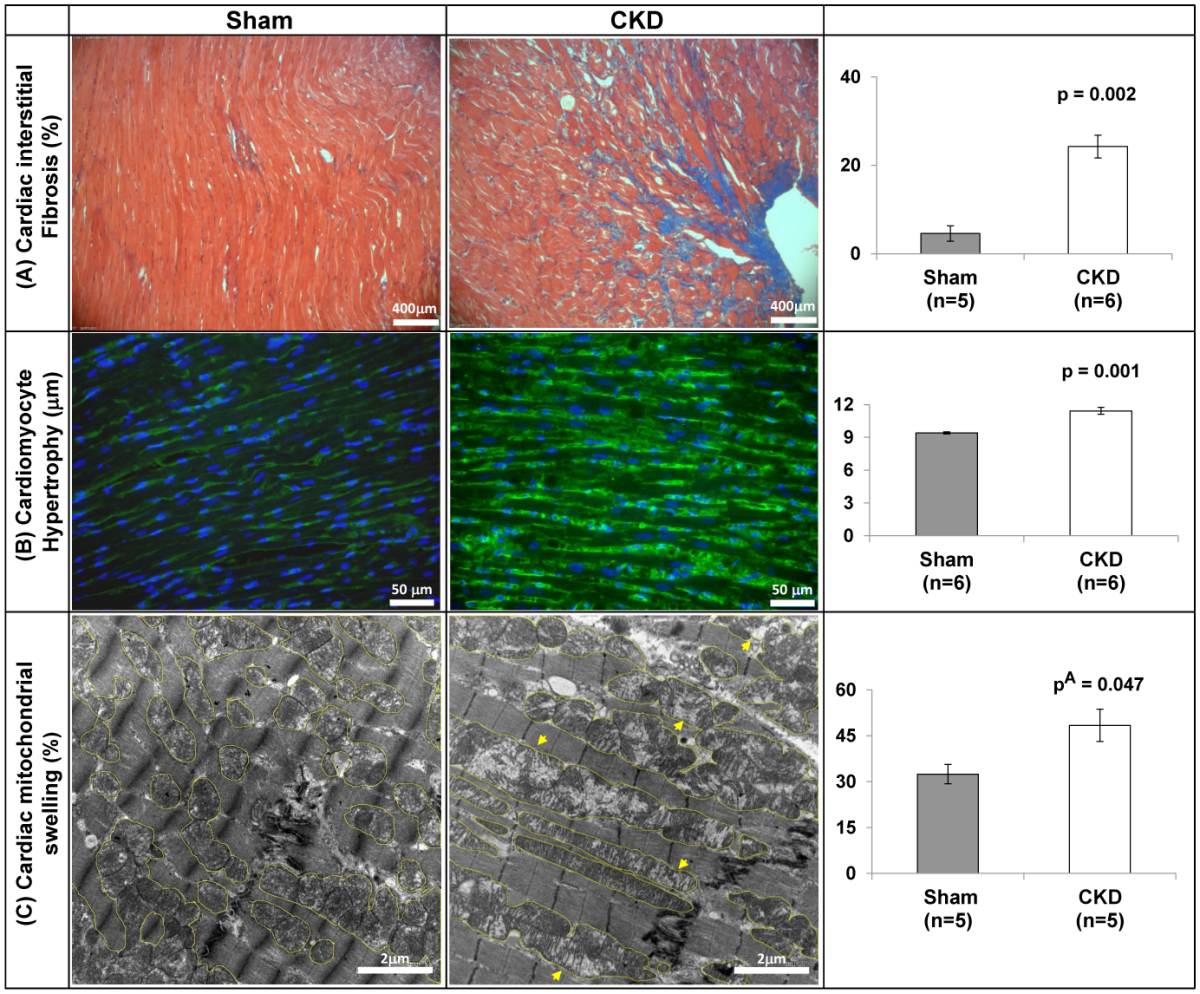
CKD setting enhances cardiac fibrosis, cardiomyocytes’ hypertrophy and cardiac mitochondrial swelling. **(A)** Representative Masson’s trichrome captures of interstitial fibrosis and quantification as percent of fibrosis out of total area (%). Magnification is x20. **(B)** FITC-WGA staining to assess myocytes’ hypertrophy; Magnification is x40. **(C)** Representative TEM micrographs and quantification of the mitochondrial volume out of total area of the field (%). Yellow circles mark the mitochondrial area and arrow heads mark swollen-deranged mitochondria. Magnification is x12K. Quantification analysis was derived from 4 fields per slides; 5–6 animals/group. Data are represented as mean ± SEM. p^A^ is based on Mann-Whitney test, otherwise p-value is based on *two-tailed t-test*.

Masson’s trichrome staining showed a significant increase in interstitial fibrosis of cardiac sections of CKD rats compared to Sham (24.3±2.55% versus 4.6±1.73%, p<0.001; Fig 2A). Likewise, WGA fluorescent staining of the cardiomyocytes’ membrane demonstrated an increased myocytes’ thickness in the CKD setting (11.42±0.31μm compared to 9.41±0.1μm, respectively, p = 0.001; Fig 2B). The histological assessment was consistent with the echocardiography findings, pointing to cardiac remodeling in CKD hearts (Table 1).

### Mitochondrial involvement in cardiac remodeling

Using TEM analysis, we aimed to explore the spatial and sub-cellular organization of the cardiac tissue of CKD animals. Surprisingly, TEM analysis revealed a massive spatial disarrangement of the muscle fiber and its organelles in the CKD group. In particular, swollen-damaged cardiac mitochondria with significantly increased volume and reduced cristae density were observed in CKD hearts; i.e. the percentage of mitochondrial volume out of total area was 48%±5.9 in CKD relative to 32%±3.2 in sham (**p**^**A**^ = 0.047; Fig 2C). A higher magnification of representative TEM images of cardiac mitochondria in Sham and CKD animals is given in supporting information figure S1 Fig.

### AngII as a potential mediator for cardiac mitochondrial damage in CKD

In light of the observed cardiac mitochondrial disarrangement of CKD rats and the modified expression of AngII in their plasma compared to control, we hypothesized that this hormone is involved in CKD-mediated damage to cardiac mitochondria. In order to test this hypothesis, short-term (11 weeks) CKD rats, that were documented with higher levels of plasma AngII compare to control (1.53±0.09pg/ml versus 0.69±0.04pg/ml, n = 6/group, p<0.0001; data not shown), were treated with Losartan, an AngII receptor blocker (ARB).

Characterization of renal, cardiac and hemodynamic parameters of the short-term CKD model confirmed the reduced body weight and Ccr, increased BUN, elevated blood pressure, increased kidney/body weight (Table 2) as well as increased in cardiac hypertrophy parameters compared to sham (Table 3), similar to the data observed in the long-term model (Table 1). While having no effect on kidney weight, body weight, Ccr, BUN or albumin excretion rate, ARB treatment partly attenuated the elevated BP in ARB-treated CKD group compared to untreated CKD animals (135±1.3/107±3.3mmHg versus 125±4.6/96±2.2mmHg; Systolic BP had a trend towards significant, p = 0.086; P-value of diastolic BP was p<0.05). Additional computation of Ccr, Urinary albumin excretion rate, Urinary protein excretion rate and LV mass per body weight is given in supporting information S3 Table.

**Table 2:** Hemodynamic and renal parameters in short-term CKD model with or without treatment with ARB.

	Sham	CKD	ARB
**Blood pressure**			
Systolic blood pressure (mmHg)	120±1.1	**135±1.3**†	**125±4.6** ^**T2**^
Diastolic blood pressure (mmHg)	93±2.2	**107±3.3** ††^A^	**96±2.2** ¥ ^A^
**Renal parameters**			
Body weight (gr)	408±15	**332±6** †††	**327±10** †††
Kidney weight (gr)	1.2±0.03	1.1±0.04	1.1±0.1
Kidney/Body weight (mg/gr)	2.9±0.1	**3.2±0.08** ^A^	3. 0±0.12 ^A^
Plasma BUN (mg/dL)	15±0.81	**58±1.18** ^**T1**^	**60±4.68** ††
Creatinine clearance (ml/min)	6.48±1.29	**0.92±0.16** †††	**0.84±0.27** †††
Urinary albumin excretion rate (mg/min)	0.04±0.01	0.21±0.19 ^A^	0.12±0.06 ^A^
Urinary protein excretion rate (mg/min)	0.59±0.05	0.80±0.1	0.83±0.08

BUN- blood urea nitrogen; ARB–CKD animals treated with ARB. P-value of CKD or ARB vs. Sham:

^**†**^ *P*<0.05,

^**††**^ *P*<0.01,

^**†††**^ P<0.001. P-value of CKD vs. ARB:

^**¥**^ P<0.05. Trend of CKD or ARB vs. Sham–T1. Trend of CKD vs. ARB–T2.

^A^ is based on Kruskal-Wallis test, otherwise p-value is based on one way ANOVA.

**Table 3:** Cardiac parameters in short-term ARB-treated model.

	Sham	CKD	ARB
**Cardiac parameters**			
LV mass (mg)	832±13	873±36	**699±34** †,¥¥
Endocardial FAC (%)	61.2±5.2	68.2±2.1	63.6±1.4
Stroke volume (μl)	227±35.9	253±22.3	219±13.9
Heart rate (bpm)	360±10.4	391±13.6	383±3.9
Ejection fraction (EF, %)	67.7±3.5	72.6±2.2	67.7±0.9
Fractional shortening (FS, %)	39.34±2.8	43.30±2.19	39.56±0.73
Intraventricular Septum Diastole (mm)	1.77±0.2	1.93±0.06	**1.51±0.03** ^**T1**^,¥¥
LV Posterior Wall Thickness in Diastole (mm)	1.64±0.1	1.84±0.07	**1.55±0.04** ¥¥
LV end diastolic diameter (LVEDD, mm)	8.13±0.59	7.62±0.17	7.93±0.18
LV end systolic diameter (LVESD, mm)	4.96±0.55	4.31±0.14 ^A^	4.79±0.09 ^A^

LV–left ventricle; FAC-fractional area change; bpm-beats per minute;

ARB–CKD animals treated with ARB. P-value of CKD or ARB vs. Sham:

^**†**^ *P*<0.05.

P-value of CKD vs. ARB:

^**¥¥**^ P<0.01. Trend of CKD or ARB vs. Sham–T1.

^A^ is based on Kruskal Wallis test, otherwise p-value is based on one way ANOVA.

Moreover, Table 3 demonstrates a reduction in the following LV hypertrophic parameters in CKD rats following treatment with ARB: LV mass, Intraventricular Septum Diastole and LV Posterior Wall Thickness in Diastole. The data confirm the established effects of ARB treatment on BP and LV structure.[24, 25]

**Fig 3:**
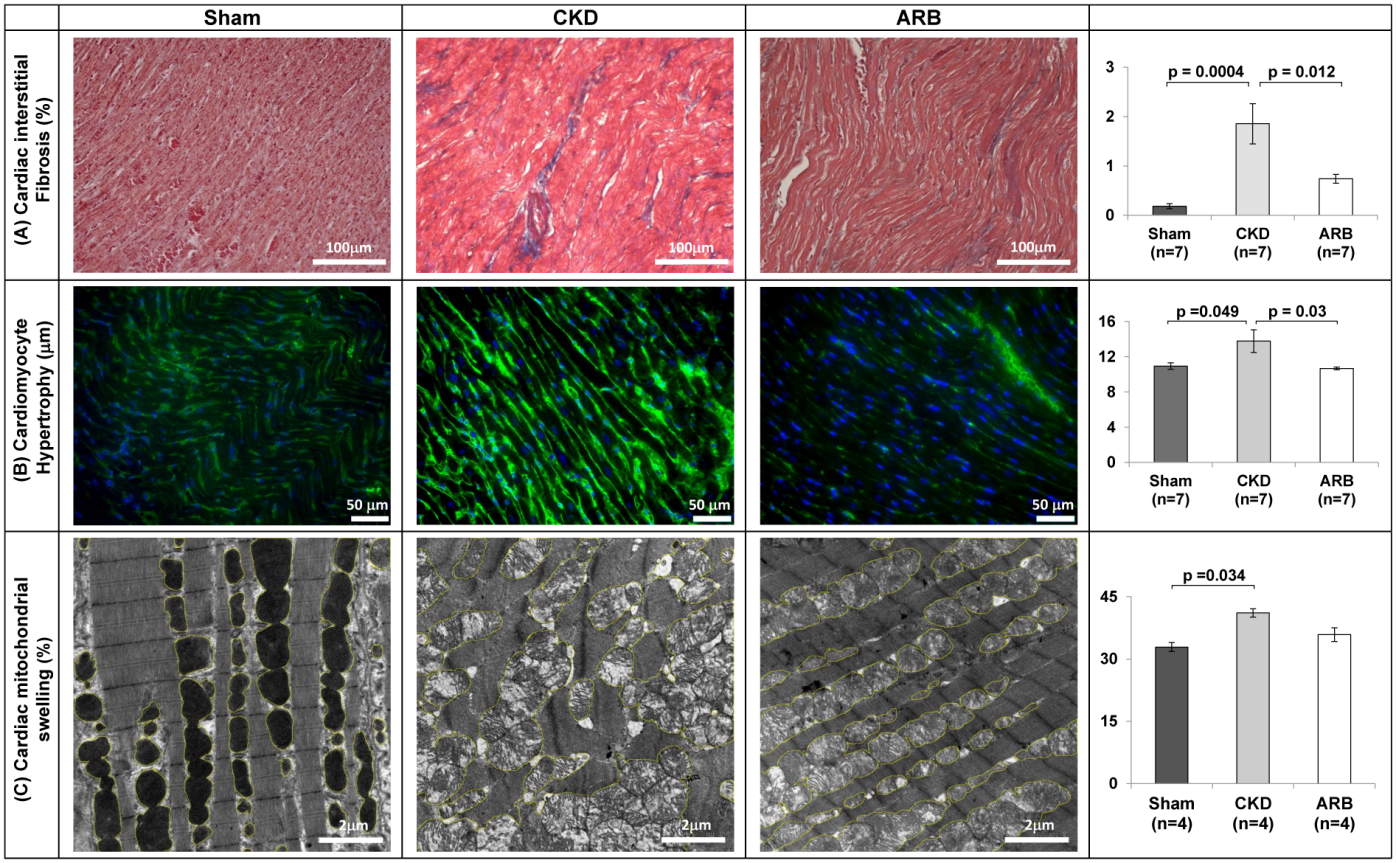
ARB treatment reduces cardiac interstitial fibrosis, cardiomyocytes’ hypertrophy and total volume of the cardiac mitochondria. **(A)** Representative Masson’s trichrome captures of interstitial fibrosis (%). Magnification X20. **(B)** FITC-WGA staining to assess myocyte hypertrophy (μm). Magnification X40. **(C)** Representative TEM micrographs as well as quantification of the mitochondrial volume out of total area of the field (%). Yellow circles mark the mitochondrial area and arrow heads mark swollen-deranged mitochondria. Magnification is x12K. Quantification was derived from 4 fields per slides; 4–7 animals/group. ARB–CKD animals treated with ARB. Data are represented as mean ± SEM. * *P*<0.05.

Next, we examined whether ARB treatment affects the extent of interstitial fibrosis, cardiomyocytes’ thickness and spatial organization of cardiac mitochondria. Indeed, we observed partial reduction in cardiac interstitial fibrosis in CKD-ARB animals: 0.74±0.09% compared to 1.85±0.4% in untreated CKD and 0.19±0.05% in sham (n = 7/group; Fig 3A). In parallel, ARB treatment reduced the cellular hypertrophy observed by WGA staining from 13.8±1.3μm in untreated CKD group to 10.7±0.16μm in ARB-treated CKD animals, similar to the values observed in the sham-operated controls animals: 10.95±0.37μm (Fig 3B). Likewise, as documented in Fig 3C, ARB administration reversed the sub-cellular disorganization of the cardiac tissue and partially reduced the increased mitochondrial volume observed at the CKD setting from 40.6±0.9% to 35.3±1.6%, relative to 32.7±1.06% in sham rats (n = 4/arm). The data suggest that AngII may have an important role in regulation of cardiac morphology in general and in particular, mitochondrial morphology.

### Molecular pathways involved in the CKD-mediated damage to cardiac mitochondria

**Fig 4:**
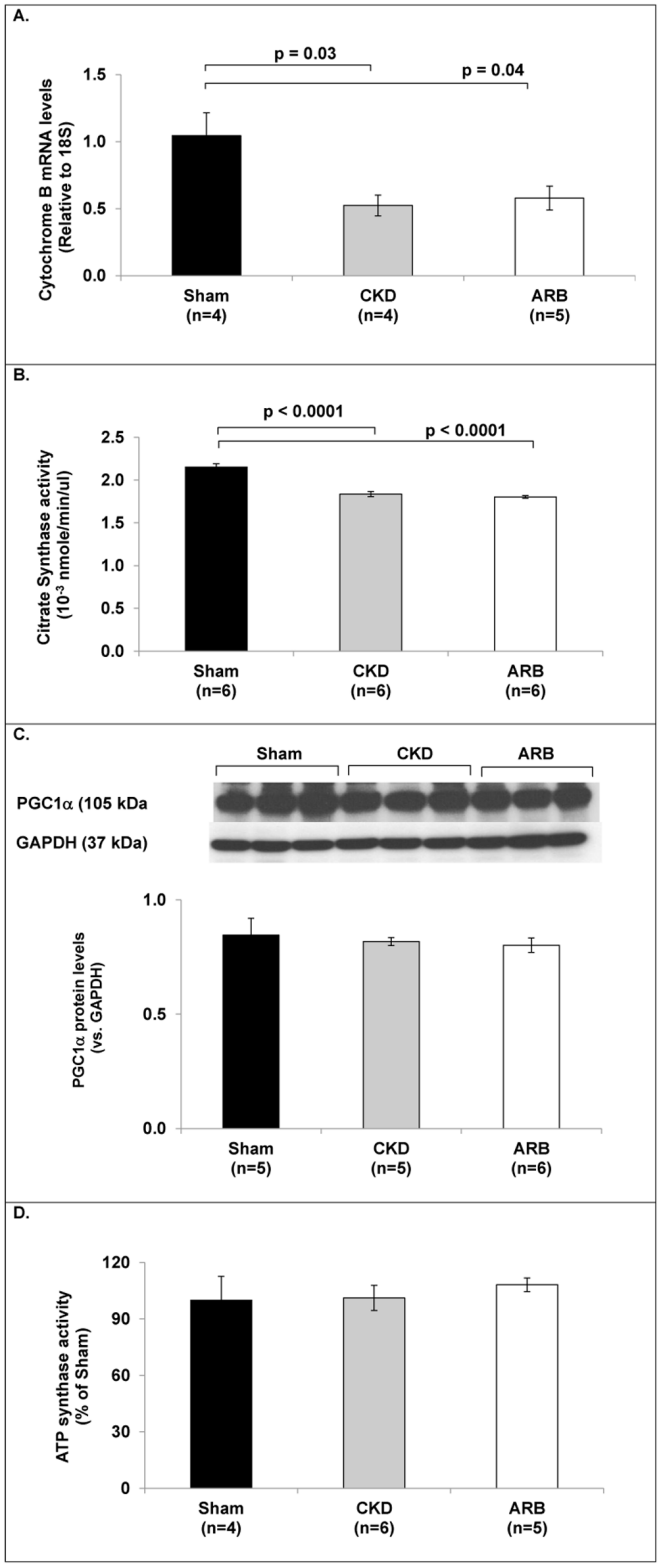
CKD-induced mitochondrial damage. **(A)** Relative expression of CytB as a marker for total mitochondrial content, relative to 18S. **(B)** Citrate synthase activity as an indicator for intact mitochondrial content. **(C)** Protein expression of PGC1α as a marker for mitochondrial biogenesis, normalized to GAPDH. **(D)** ATP synthase (complex V) activity. All data are given for the 3 experimental groups: sham, CKD and ARB (4–6 animals/group). ARB–CKD animals treated with ARB. Data are represented as mean ± SEM.

Utilizing the short-term CKD model, we performed several assays aiming to explore the molecular pathways involved in the CKD-mediated damage to cardiac mitochondria with or without treatment with ARB. First we assessed cardiac mitochondria content using real-time PCR analysis of the mitochondrial encoded gene Cytochrome B (CytB), an accepted marker for mitochondrial DNA content (mtDNA).[26] CytB gene expression was decreased by 48% in CKD rats and by 42% in ARB-treated CKD rats compared to Sham (from 1.04±0.17 in Sham to 0.52±0.07 in CKD rats, p = 0.03; or 0.58±0.09 in ARB rats, p = 0.04; n = 4-5/arm). No significant differences were observed in CytB expression between untreated CKD and ARB-treated CKD groups (Fig 4A). Citrate synthase activity, a ubiquitous marker for mitochondrial content,[27] was significantly reduced in the CKD rats, pointing to reduced number of intact mitochondria in this model (1.83±0.03x10^-3^ nmole/min/μl in CKD versus 2.15±0.03x10^-3^nmole/min/μl in sham animals, p<0.0001). ARB treatment failed to increase CS activity (1.8±0.02x10^-3^nmole/min/μl, p = 0.7; n = 6/arm; Fig 4B). The specific activity of ATP synthase (% of Sham) did not change among the three groups (Sham 100±12%, CKD 101±6%, CKD-ARB 108±3%; Fig 4D). These data may indicate that while part of the mitochondria undergoes degradation, the intact ones preserve normal activity of the respiratory chain. Likewise, despite the marked decrease in cardiac mitochondrial content (Fig 4A), no differences were observed in the protein levels of the transcription factor for mitochondrial biogenesis, PGC1α,[28] between sham, CKD or CKD-ARB groups (Fig 4C). The data imply that no compensatory response to generate new cardiac mitochondria takes place.

**Fig 5:**
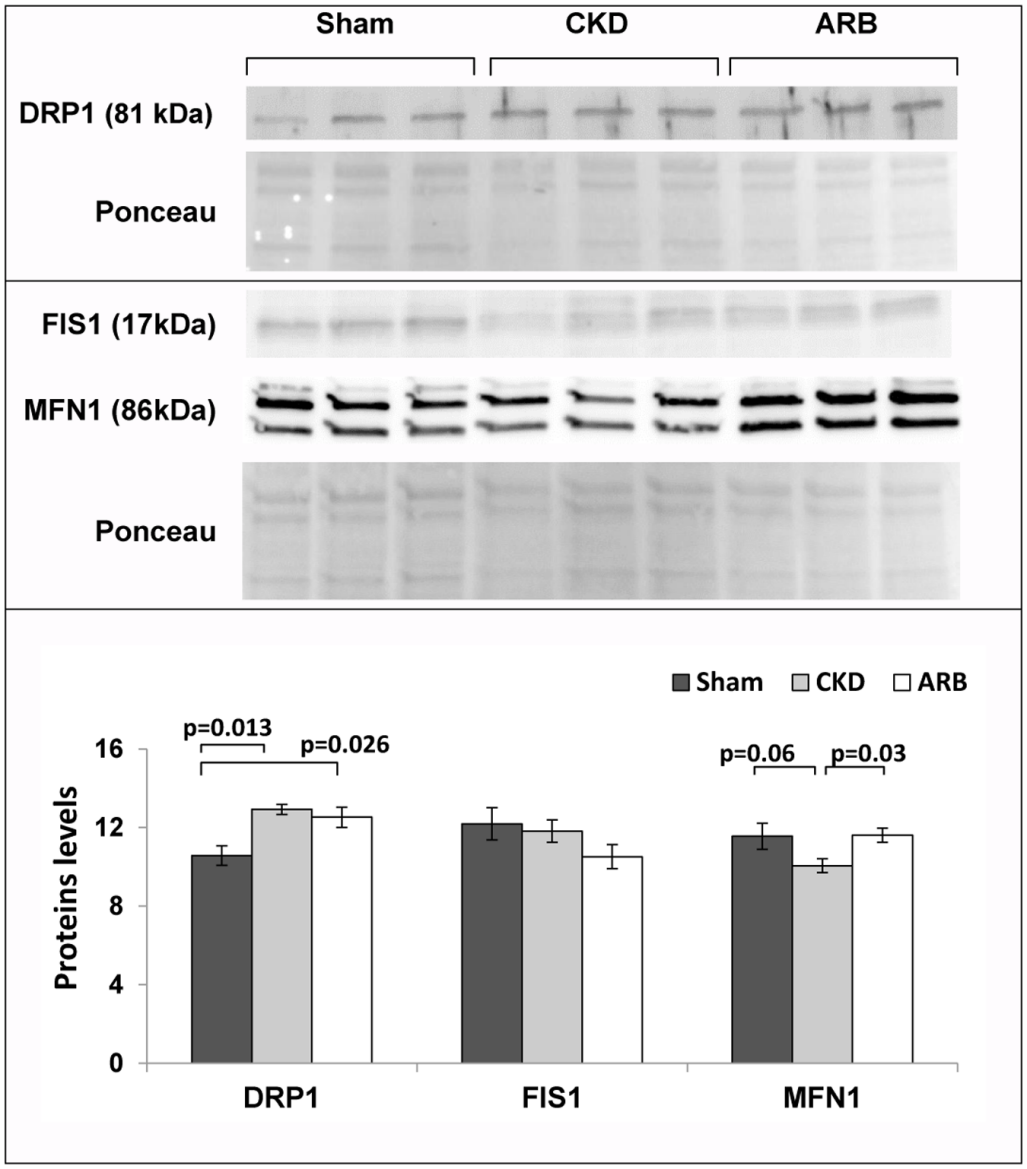
Mitochondrial biogenesis and quality control. Protein expression of the fission protein MFN1 as well as the fusion proteins FIS1 and DRP1. N = 4–6 animals/group. ARB–CKD animals treated with ARB. Data are represented as mean ± SEM.

In order to evaluate whether the pathological presentation of cardiac mitochondria in the CKD setting stems from alteration in mitochondrial quality control mechanisms, we aimed to assess the expression levels of proteins involved in known quality control mechanisms of the mitochondria, i.e. fission and fusion. To this end we tested the expression levels of the known fusion protein MFN1 as well as two fission proteins FIS1 and DRP1 in cardiac lysates of the three experimental groups. Interestingly, as presented in Fig 5, we observed a mild over-expression of the fission protein DRP1 in the CKD group relative to control (12.9±0.26 versus 10.6±0.49; p = 0.013); but no differences in FIS1 protein levels (11.82±0.57 versus 12.19±0.81; p = 0.92). ARB treatment did not affect DRP1 (12.5±0.5; p = 0.8), and FIS1 expression (10.8±0.61, p = 0.34). In addition, a small reduction in MFN1 expression with a trend towards significance compared to sham was observed in CKD lysates (9.8±0.3 versus 11.6±0.6; p = 0.064). ARB treatment reversed the reduction of MFN-1 levels similar to control levels compared to CKD group (12±0.47; p = 0.029). Altogether the data suggest that only a minor shift in the balanced processes of cardiac mitochondrial quality control takes place in the CKD setting, and that this minor shift is partially reversed by ARB treatment.

**Fig 6:**
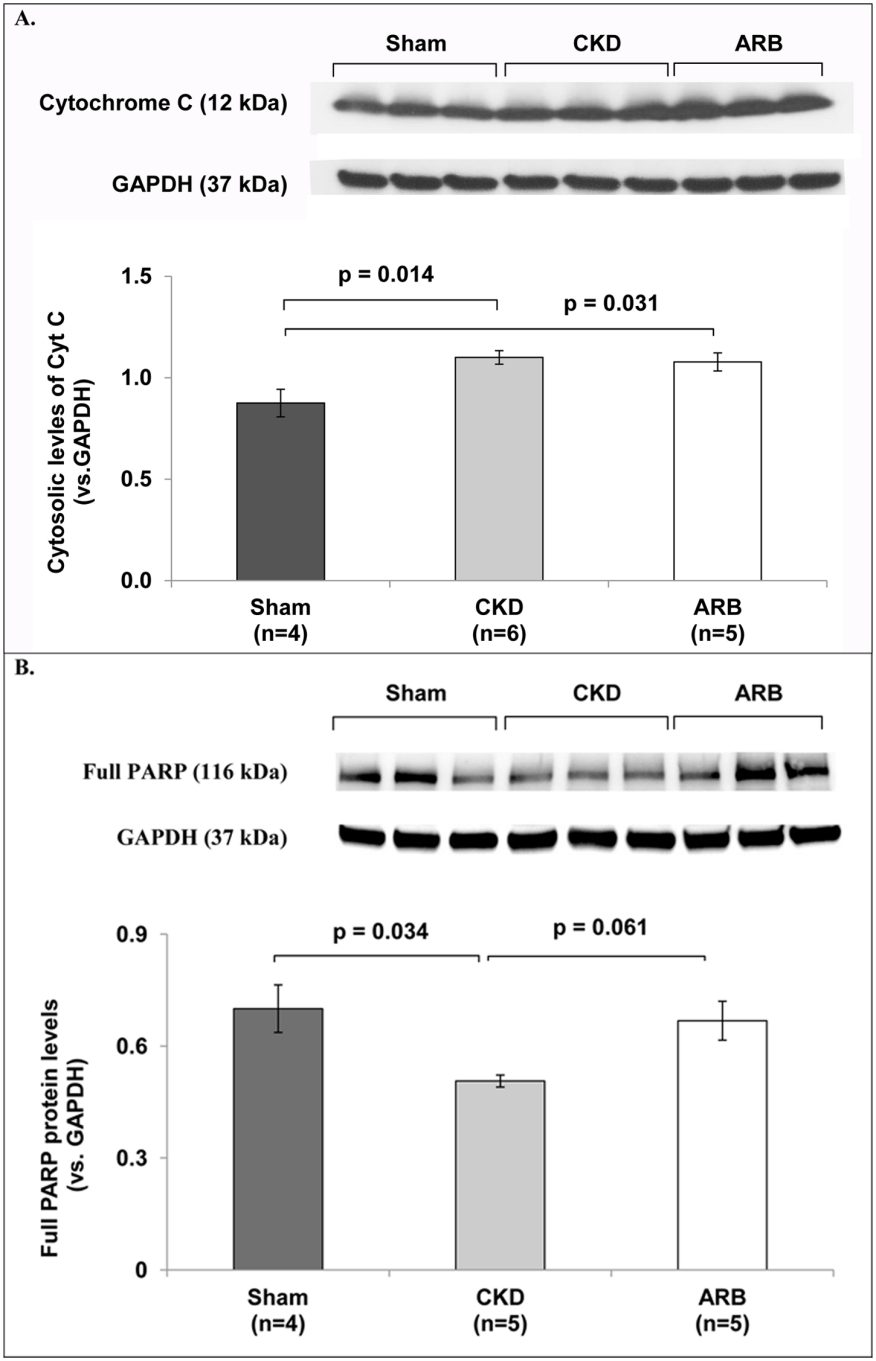
Potential mechanisms for CKD-induced mitochondrial damage. **(A)** Relative CytC expression (CytC leakage) in cytosolic fractions of cardiac cells. **(B)** Protein expression of PARP-1 as a marker for cellular apoptosis, normalized to GAPDH. N = 4–6 animals/group. ARB–CKD animals treated with ARB. Data are represented as mean ± SEM.

In view of the pathological presentation of swollen cardiac mitochondria that occurs concomitantly with cardiac hypertrophy and hardly involves mitochondrial quality control mechanisms, we hypothesized that the underlying mechanism for this observation involves cardiac cell death and mitochondrial degradation. In order to test this hypothesis we have measured the expression levels of several markers in the three experimental arms. The markers included cytosolic CytC- a marker for structural damage to the mitochondrial membrane which is normally located at the mitochondrial intermembrane space; LC3- marker for cellular autophagy; Pink-1- mitophagy marker and PARP-1 cleavage- a hallmark for cellular apoptosis. Western analysis for CytC levels in the cytosolic fractions of cardiac lysates indicated a mild cytosolic accumulation of CytC in CKD hearts relative to sham-operated controls (1.1±0.03 versus 0.87±0.07; p = 0.014; Fig 6A). ARB treatment did not affect cytosolic levels of CytC compared to untreated CKD group (1.08±0.04; p = 0.9). Despite increased levels of cytosolic CytC, the data obtained did not show any marked elevation in the protein marker for autophagy, LC3 or the mitophagy marker Pink-1,[29] in CKD or CKD- ARB compared to sham hearts (data not shown). The data imply that the leakage through the mitochondrial membrane *per-se* in the CKD setting does not culminate in mitophagy or cardiomyocytes’ autophagy. Nevertheless, in line with the increased mitochondrial damage reflected by CytC leakage to the cytosol, the data presented in Fig 6B point to increased cellular apoptosis in the CKD setting as reflected by the reduced levels of full-length PARP-1, a hallmarked for cellular apoptosis (from 0.7±0.06 of sham to 0.5±0.03 of CKD, p = 0.034). Interestingly, treatment with ARB fully attenuated PARP-1 cleavage (0.67±0.11, p = 0.061 compared to CKD). Altogether the data suggest that the pathological presentation of swollen cardiac mitochondria mainly stems from CKD-mediated cardiac remodeling processes that lead to cardiac cell death and mitochondrial damage.

## Discussion

It is currently recognized that CKD can often manifest in LV remodeling and hypertrophy, which may culminate in LV dysfunction and heart failure.[6] Renal histology of CKD animals performed in a previous study of ours has demonstrated a significant hypertrophy of the proximal tubules, increased interstitial fibrosis as well as interstitial inflammation.[16] The current study was designed in order to test the hypothesis that CKD results in cardiac pathology, with special attention to cardiac mitochondria and the involvement of AngII- a hallmark of the Renin Angiotensin System (RAS) [4], in the underling processes. Indeed, we have identified a moderate but statistically significant increase in AngII levels in the plasma of CKD animals versus sham-operated controls. We thus anticipate that RAS may represent one of the key players in the CKD-mediated cardiac hypertrophy and mitochondrial structural disorganization.

We have postulated that mitochondria, which hold crucial importance in the heart and were implicated in numerous cardiovascular diseases,[10–15] may be damaged in cardiac sections of CKD animals and be at least in part, salvaged by application of an AngII blocker.

Indeed, we have documented swollen-damaged highly disorganized mitochondria in the hearts of CKD-induced rats, concomitantly to enhanced cardiac hypertrophy in general and cardiomyoctes’ hypertrophy in particular as well as interstitial fibrosis. Surprisingly, this pathological presentation was documented even though the systolic function of the hypertrophic LV has not yet deteriorated. These data stand in line with our previous GeneChip array data which suggest that the two pathological processes of cardiac hypertrophy and mitochondrial damage occur shortly after CKD induction.[18]

The increased volume of cardiac mitochondria was associated with reduction of total as well as intact mitochondrial content, and with CytC leakage to the cytosol and with PARP-1cleavage which is associated with enhanced cellular apoptosis. However, we have not observed any changes in autophagic activity represented by the autophagy marker LC3B or the mitophagy marker Pink-1. The data support that cardiac hypertrophy accompanied by mitochondrial damage, precedes any cardiac dysfunction, as seen in echocardiography. Both mitochondrial fission and fusion processes are known to affect the morphology and functionality of the mitochondria. These processes are well balanced in physiological conditions but may alter in diseases.[30–34] Indeed, we have documented a minor induction in the expression of the fission-related protein DRP1 as well as a minor reduction in the level of the fusion protein MFN1, suggesting some mitochondrial fragmentation in the CKD setting, which is partially salvaged by ARB. Nevertheless, we have noted that the total volume of cardiac mitochondria as measured in the TEM analysis was increased in CKD relative to sham, potentially due to mitochondrial swelling which is evident by the non- condensed cristae. Thus, in line with the data pointing to mitochondrial damage and cellular apoptosis, we anticipate that the increased mitochondrial volume represents mitochondrial swelling that occurs during the pathological process of cardiac hypertrophy, rather than mitochondrial fission. Moreover, we have not documented any modified expression of the *de-novo* mitochondrial biosynthesis marker, PGC1α or in ATP synthase specific activity. Thus, altogether the data highly suggest that the pathological appearance of cardiac mitochondria mainly stems from CKD-mediated cardiac remodeling processes rather than from mitochondrial-oriented cell death or from major mitochondrial quality control processes.

It is worthwhile to note that the pro-inflammatory response, is known to be increased and to positively correlate with the severity of the cardiac disease [1–3]; It has been recently suggested that pro-inflammatory mediators are able to upregulate RAS and that the interplay between TNFα and AngII is mediated by increased production of reactive oxygen species (ROS) through NOX pathways.[35] In the current study we have identified modified expression of pro-inflammatory cytokines (IL-1α and TNFα) and the anti-inflammatory agent IL-10. The concomitant induction of IL-10 in parallel to the induction of the pro-inflammatory cytokines is somewhat surprising. Yet, it has recently been reported that inflammatory agents as well as the anti-inflammatory cytokine IL-10 are increased in acute-coronary syndrome-induced CRS.[36] The authors anticipated that elevated sera levels of IL-10 may represent a counter action to the heightened inflammatory state in CRS.

As expected, ARB treatment partly attenuated blood pressure, markedly attenuated LV mass, and significantly reduced cardiac hypertrophy and fibrosis. Interestingly, our data indicate that ARB administration partially attenuated cardiac mitochondrial swelling. Yet, despite the prominent beneficial effects of ARB on the spatial organization and relative volume of cardiac mitochondria in the CKD state, ARB failed to increase the content of total or intact mitochondria and had no to little effect only on mitochondrial quality control mechanisms. The data thus suggest that ARB may not act directly on cardiac mitochondria; the observed beneficial outcome of ARB treatment on the mitochondrial structure may be secondary to its well-recognized effects on reducing cardiac hypertrophy.[37, 38]

The data presented herein highly suggest that cardiac mitochondria alterations in CKD conditions precede cardiac dysfunction that does not occur in early stage of CKD. In line with our observations of mitochondrial membrane damage and induced cardiac apoptosis, a previous study, conducted in CKD rats, has pointed to increased cardiac oxidative stress, a process which is tightly linked with mitochondrial dysfunction.[39] The data thus suggest that cardiac mitochondria may represent an important target for treating not only patients with heart failure or cardiomyopathy, but also CKD patients with not yet apparent cardiac dysfunction, with the ultimate goal of attenuating progression to CRS.

**Fig 7:**
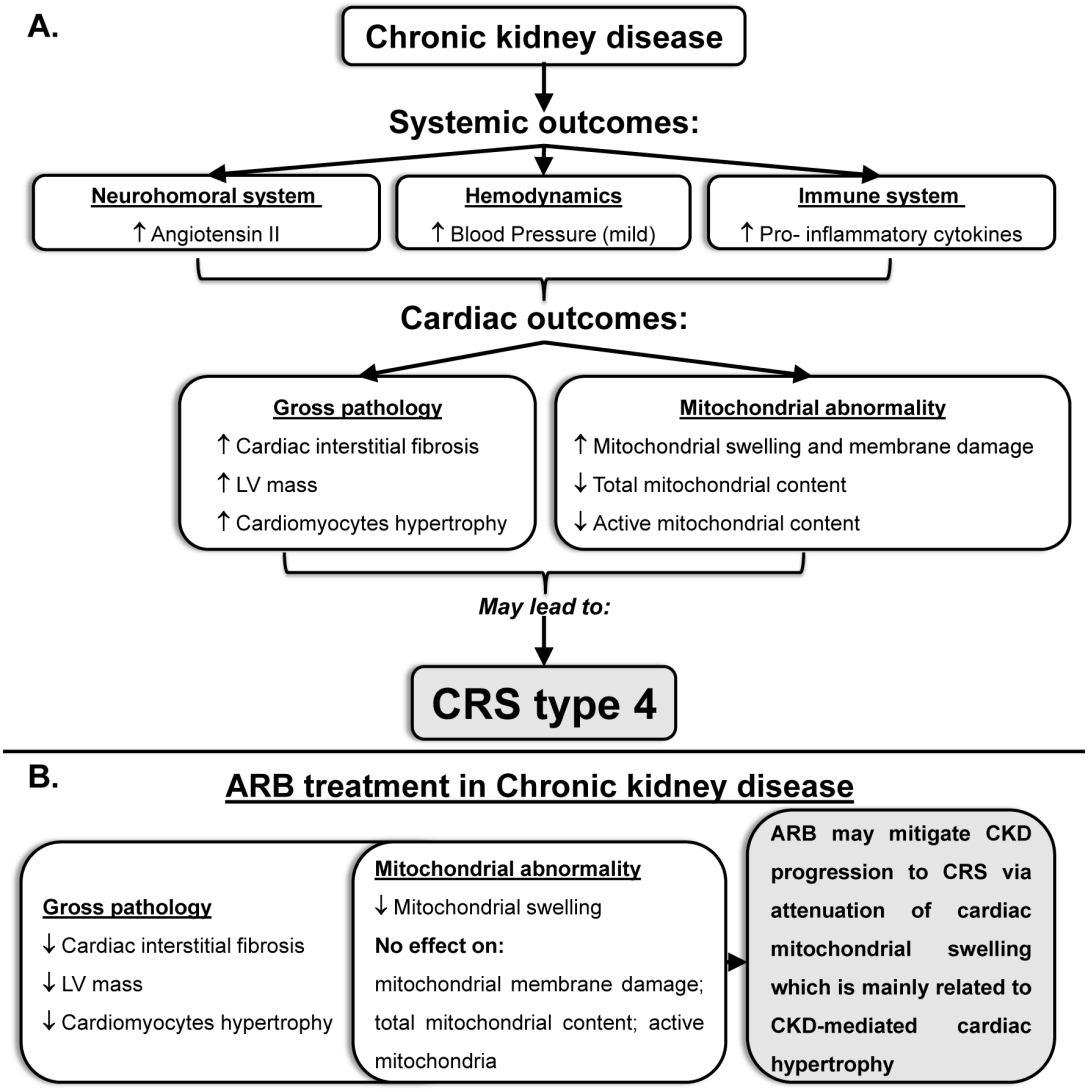
A potential kidney-heart interplay which may mediate changes in mitochondrial structure and function in CKD. **(A)** CKD setting results in cardiac gross pathology (manifested by increased fibrosis, LV mass and cellular hypertrophy) as well as mitochondrial abnormality (manifested by mitochondrial swelling, decreased mitochondrial content and active mitochondrial content) through mild elevation in blood pressure and activation of neurohumoral and immune systems. These changes may lead to the progression of CKD to CRS type 4. **(B)** ARB treatment mainly attenuates changes in the gross pathology of the heart as well as mitochondrial abnormalities.

A potential scheme for the kidney-heart interplay which may mediate changes in the mitochondrial structure and function in CKD with or without treatment with ARB is given Fig 7. ARB treatment may mitigate CKD progression to CRS via attenuation of cardiac mitochondrial swelling which is mainly related to CKD-mediated cardiac hypertrophy.

It is worthwhile to note that blood pressure was somewhat elevated in CKD group relative to sham and was reduced to normal level by ARB. Nevertheless, since hypertension in rats is defined by BP of at least 150/90,[28, 40, 41] we considered the CKD rats, which had significantly lower BP, as non-hypertensive. Thus, we believe that the changes of cardiac mitochondria are merely attributed to the systemic effects CKD condition *per-se* rather to hypertension. Yet, in order to completely exclude potential effects of elevated BP, an additional *in vivo* experiment in which hypertension will be induced by AngII infusion without CKD induction [42] may be warranted. This additional experiment may enable us to carefully compare between the potential effects of CKD versus hypertension *per-se* on the structure and function of cardiac mitochondria.

### Study limitations

Even though our prolong CKD model resulted in increased myocardial fibrosis and highly significant mitochondrial dysfunction, overt systolic heart failure was not documented. Moreover, at heart rates of 330–450 bpm, we were unable to measure reliably diastolic parameters by Doppler echocardiography, since under these conditions LV filling time is significantly shortened; on the other hand, reducing heart rate by applying deeper anesthesia may misleadingly present as reduced systolic LV function. Therefore we were unable to determine whether or not the CKD rats experience diastolic dysfunction under our available technology. A direct measurement of systolic or diastolic dysfunction can be performed by utilizing pressure-volume loops at the experimental endpoints; this procedure will be employed in our subsequent studies.

## Conclusions

Impaired cardiac mitochondrial structure combined with increased mitochondrial swelling may represent a crucial risk factor for developing cardiovascular complications in the CKD setting. This pathological presentation could be partially reversed by blockade of the AngII signal. We believe that identifying additional circulatory mediators which may be involved in initiation and progression of cardiac mitochondrial dysfunction in CKD patients might hold pivotal importance in attenuating disease progression to the highly prevalent life threatening CRS condition.

## Supporting information

S1 Table Mitochondrial parameters of sham animals treated with ARB vs. sham-only group. (DOCX)

S2 Table Primers of mitochondrial genes of sham animals treated with ARB. (DOCX)

S3 Table Calculated parameters per 100 gr body weight. NS- non significant.
(DOCX)

S1 Fig TEM images of cardiac mitochondria in sham and CKD animals. Representative TEM micrographs of sham (A) and CKD (B) cardiac. Magnification is x30K. Scale bar 1μm.
(TIF)
