# Supplementary material for: Pathological presentation of cardiac mitochondria in a rat model for chronic kidney disease
Source: PLoS One. 2018 Jun 11;13(6):e0198196. doi: 10.1371/journal.pone.0198196 (PMC5995391; doi:10.1371/journal.pone.0198196)
Supplement: S1 Table — (DOCX) [file pone.0198196.s001.docx]

**S1 Table. Mitochondrial parameters of Sham animals treated with ARB vs. Sham-only group.**

| **Mitochondrial parameters** | **Sham** | **Sham+ARB** | **p-value** |
| --- | --- | --- | --- |
| **Cytochrome B mRNA levels (Relative to 18S)** | 1.04±0.17 | 0.77±0.12 | 0.22 |
| **PGC1α mRNA levels (vs. GAPDH)** | 1.02±0.12 | 1.1±0.22 | 0.75 |
| **ATP synthase activity (% of Sham)** | 100±12.6 | 104±21 | 0.9 |
| **Fis1 mRNA levels (vs. GAPDH)** | 1±0.03 | 1.12±0.3 | 0.72 |
| **DRP1 mRNA levels (vs. GAPDH)** | 1±0.06 | 1.17±0.26 | 0.64 |
| **OPA1 mRNA levels (vs. GAPDH)** | 1.01±0.08 | 0.86±0.1.9 | 0.45 |
| **MFN1 mRNA levels (vs. GAPDH)** | 1±0.07 | 0.8±0.19 | 0.34 |
