# Supplementary material for: Pathological presentation of cardiac mitochondria in a rat model for chronic kidney disease
Source: PLoS One. 2018 Jun 11;13(6):e0198196. doi: 10.1371/journal.pone.0198196 (PMC5995391; doi:10.1371/journal.pone.0198196)
Supplement: S2 Table — (DOCX) [file pone.0198196.s002.docx]

**S2 Table. Primers of mitochondrial genes of Sham animals treated with ARB**

| **The gene** | **Primers** |
| --- | --- |
| **Cytochrome B (CytB)** | 5'-TGACCTTCCCGCCCCATCCA-3';  5′-AGCCGTAGTTTACGTCTCGGCA-3' |
| **PGC1α** | 5'-TTCCAAAACGGATATACTCATTAACA-3';  5′- GGAGGCATCTTTGAAGTCTAGTTG-3' |
| **DRP1** | 5'- CCCGTGGATGATAAAAGTGC-3';  5'- CAAATCCTAGCACCACCACAT-3' |
| **FIS1** | 5'- GGCAACTACCGGCTCAAG-3';  5'- TTCATGGCCTTGTCAATCAG-3' |
| **OPA1** | 5'- CAGAAGACCTCGCCAATTTC-3';  5'- CTAAATGCTGTTTCTCCAGGTGT-3' |
| **MFN1** | 5'- CATCCCTCACGTCTAGAACCTC-3';  5'- CAGCCCACTGTTTTCCAAAT-3' |
| **GAPDH** | 5'-TGCACCACCAACTGCTTA-3';  5′-GGATGCAGGGATGATGTTC-3' |
| **18S** | 5'-TTGATTAAGTCCCTGCCCTTT-3';  5'-CGATCCGAGGGCCTAACTA-3' |
