# Supplementary material for: Pathological presentation of cardiac mitochondria in a rat model for chronic kidney disease
Source: PLoS One. 2018 Jun 11;13(6):e0198196. doi: 10.1371/journal.pone.0198196 (PMC5995391; doi:10.1371/journal.pone.0198196)
Supplement: S3 Table — NS- non significant. (DOCX) [file pone.0198196.s003.docx]

**S3 Table. Calculated parameters per 100 gr body weight.** NS- non significant

|  | **Sham** | **CKD** | **ARB** | **p-value** | | |
| --- | --- | --- | --- | --- | --- | --- |
| **Calculated parameters per 100 gr body weight** | | | | **Sham** | | **CKD** |
|  |  |  |  | **CKD** | **ARB** | **ARB** |
| Creatinine clearance (ml/min) | 1.43±0.26 | 0.92±0.16 | 0.84±0.27 | **0.003** | **0.002** | NS (0.99) |
| Urinary albumin excretion rate (mg/min) | 0.008±0.001 | 0.006±0.0003 | 0.043±0.023 | NS (0.99) | NS (0.37) | NS (0.26) |
| Urinary protein excretion rate (mg/min) | 0.13±0.02 | 0.22±0.03 | 0.83±0.08 | NS (0.2) | NS (0.1) | NS (0.74) |
| LV mass (mg) | 284±4.7 | 326±13.5 | 281±23 | NS (0.24) | NS (0.99) | NS (0.16) |
