# Supplementary figures and images for: Pathological presentation of cardiac mitochondria in a rat model for chronic kidney disease
Source: PLoS One. 2018 Jun 11;13(6):e0198196. doi: 10.1371/journal.pone.0198196 (PMC5995391; doi:10.1371/journal.pone.0198196)

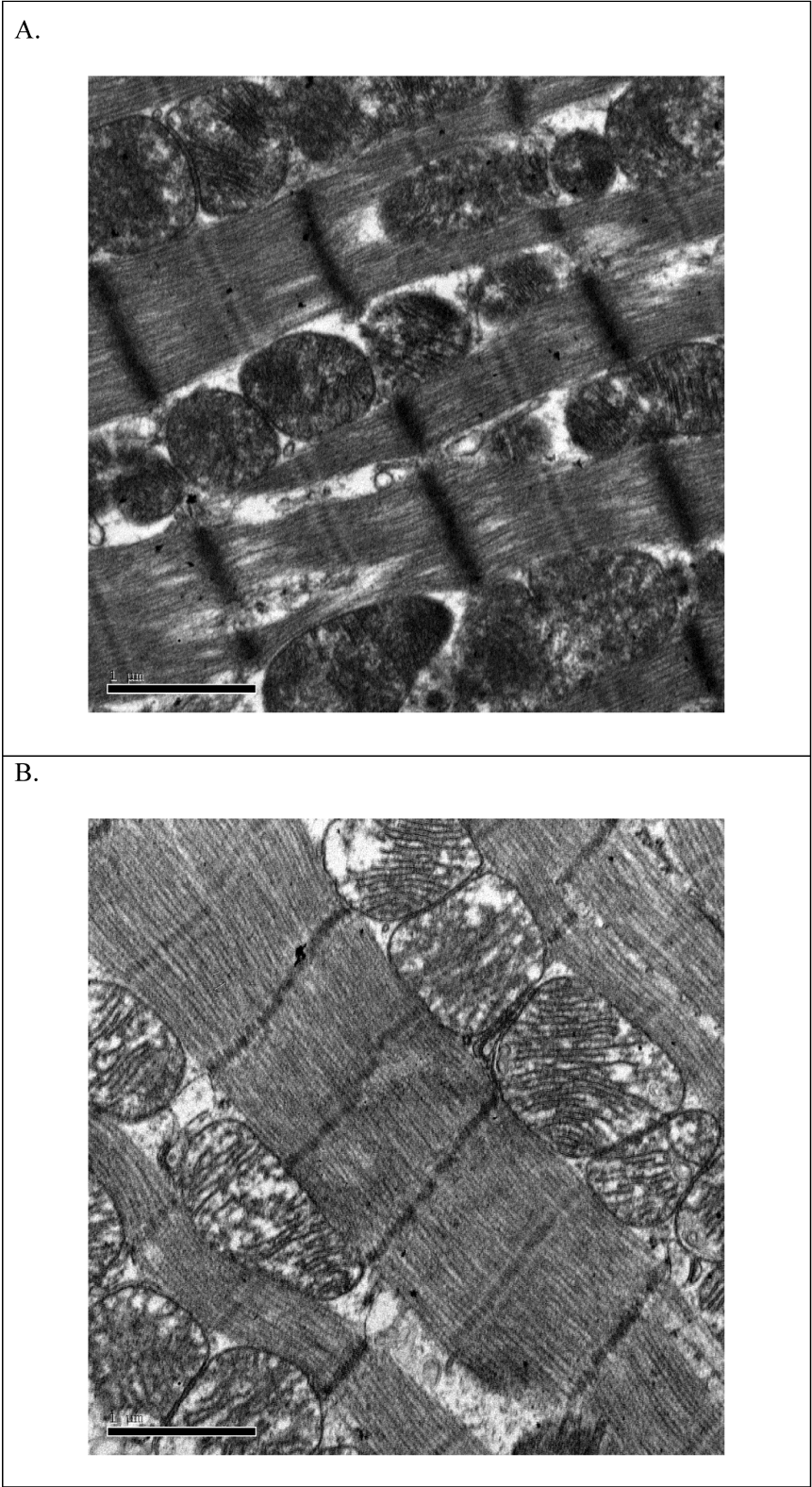

Supplement: S1 Fig — Representative TEM micrographs of sham (A) and CKD (B) cardiac. Magnification is x30K. Scale bar 1μm. (TIF) [file pone.0198196.s004.tif]
